# Inhibition of HIV Virus by Neutralizing Vhh Attached to Dual Functional Liposomes Encapsulating Dapivirine

**DOI:** 10.1186/s11671-016-1558-7

**Published:** 2016-07-28

**Authors:** Scarlet Xiaoyan Wang, Johan Michiels, Kevin K. Ariën, Roger New, Guido Vanham, Ivan Roitt

**Affiliations:** 1 Department of Natural Sciences, Middlesex University, London, UK; 2 Institute of Tropical Medicine, Antwerp, Belgium; 3 Proxima Concepts. Ltd, London, UK

**Keywords:** HIV/AIDS, Liposome, Neutralizing antibody, Drug delivery, Prophylaxis

## Abstract

Although highly active antiretroviral therapy (HAART) has greatly improved the life expectancy of HIV/AIDS patients, the treatment is not curative. It is a global challenge which fosters an urgent need to develop an effective drug or neutralizing antibody delivery approach for the prevention and treatment of this disease. Due to the low density of envelope spikes with restricted mobility present on the surface of HIV virus, which limit the antibody potency and allow virus mutation and escape from the immune system, it is important for a neutralizing antibody to form bivalent or multivalent bonds with the virus. Liposome constructs could fulfil this need due to the flexible mobility of the membrane with its attached antibodies and the capacity for drug encapsulation. In this study, we evaluated the neutralization activity of a range of liposome formulations in different sizes coated with anti-gp120 llama antibody fragments (Vhhs) conjugated via either non-covalent metal chelation or a covalent linkage. The non-covalent construct demonstrated identical binding affinity to HIV-1 envelope glycoprotein gp120 and neutralizing ability for HIV virus as free Vhh. Although covalently linked Vhh showed significant binding affinity to gp120, it unexpectedly had a lower neutralization potency. This may be due to the comparability in size of the viral and liposome particles restricting the number which can be bound to the liposome surface so involving only a fraction of the antibodies, whereas non-covalently attached antibodies dissociate from the surface after acting with gp120 and free the remainder to bind further viruses. Covalently conjugated Vhh might also trigger the cellular uptake of a liposome-virion complex. To explore the possible ability of the antibody-coated liposomes to have a further function, we encapsulated the hydrophobic antiviral drug dapivirine into both of the non-covalently and covalently conjugated liposome formulations, both of which revealed high efficacy in reducing viral replication in vitro. Thus, dual function liposomes may lead to a novel strategy for the prophylaxis of HIV/AIDS by combining the neutralizing activity of Vhh with antiviral effects of high drug concentrations.

## Background

Human immunodeficiency virus (HIV), responsible for the acquired immunodeficiency syndrome (AIDS), is a lentivirus belonging to the retrovirus family. Infection causes extensive destruction of T-helper cells, macrophages, dendritic cells and other cellular components associated with cell-mediated immunity, eventually leading to AIDS [1, 2]. As a consequence, HIV-infected patients are more susceptible to opportunistic infections [3]. At present, HIV-1 and HIV-2 are the two known types of HIV [4]. HIV-1 is more pathogenic and transmissible and is mainly responsible for the global AIDS pandemic [4]. HIV infection has continued spreading by the transfer of body fluids due to exposure to blood or blood products, by homo- or heterosexual contact, or prenatally from mother-to-child. Since the start of the epidemic, around 78 million people have become infected with HIV and 35 million people have died of AIDS-related illnesses according to the global statistical report from UNAIDS in 2016. In addition, 2.1 million people became newly infected with HIV and 36.7 million people were living with HIV globally in 2015.

Currently, the major means of efficient management of HIV and prevention of its progression towards AIDS is highly active antiretroviral therapy (HAART), which combines a minimum of three antiretroviral drugs from at least two classes. These include nucleoside reverse transcriptase inhibitors (NRTIs), non-nucleoside reverse transcriptase inhibitors (NNRTIs), protease inhibitors (PIs), fusion inhibitor, integrase inhibitors and/or entry inhibitors [5]. Although the therapy has improved long-term survival rate, there are associated toxic side effects and emergence of multi-drug resistance. Furthermore, there is a limited access to anti-HIV drugs in the developing countries due to their high costs [6]. Clearly, there remains an urgent demand for an effective and low-cost system for prophylaxis and treatment of HIV infection.

The challenge of developing an effective vaccine or entry-inhibitor drug for HIV infection lies in the rapid mutation of the glycoproteins comprising gp120 and gp41 and the structural features that facilitate antibody evasion [7–13]. Another recent explanation is the spatial arrangement of the envelope spikes on HIV virus, which contributes to its ability to escape the neutralization by antibodies [14]. As is known, most neutralization activities can be attributed to antibody-antigen interactions and the avidity resulting from bivalent binding between two Fabs and two physically linked antigens greatly increase the neutralization potency of the antibody [15–17]. The published studies indicated that there are 4 to 35 spikes per HIV virus particle [18–22] with very restricted mobility [23, 24]. This makes the antibody cross-linking very unlikely because the hinge (10–15 nm) between the two Fabs is not long enough to reach the two spikes randomly distributed on the surface of the virus. When the anti-HIV antibody b12 was tested as an IgA, IgM or IgG, equivalent neutralization potency was observed [14]. Actually, a number of studies have demonstrated that neutralizing antibodies against HIV cannot take the advantage of avidity effects resulting from the IgG cross-linking epitopes on the surface of virus [15–17], while antibodies have been shown to bind bivalently to other viruses comprising closely spaced epitopes [25, 26].

Liposomes are artificially prepared vesicles composed of a lipid bilayer and have been used extensively for drug delivery due to their unique properties [27]. Liposomes are also platforms for the development of multivalent antibody constructs by coupling antibody molecules onto liposome membranes. Chiu et al. have demonstrated that the antibody potency was increased 25-fold by employing the multivalent liposome formulation [28], which even induced cascade effects by cross-linking the antibody complex [29].

Recently, llama heavy-chain antibody fragments (Vhh), which compete with surface CD4 for binding to the HIV-1envelope glycoprotein, have been described as potent cross-clade HIV-1 candidate microbicides [30, 31]. Such Vhh would be excellent candidates for multivalent liposome platform due to their specificity, high stability and low production cost [31, 32]. Moreover, these antibody fragments showed very efficient ability to penetrate human tissue by diffusing into the mucosal layer when placed in a vaginal ring [31].

In this study, multivalent liposomal antibody constructs were developed by grafting Vhh (J3), which achieves broad and potent neutralization of HIV-1 via interaction with the CD4-binding site of HIV-1 env [33], onto the liposome membrane via either non-covalent metal chelation or a covalent linkage. The neutralization potency of multivalent presentation of these fragments by liposomes was analyzed by antiviral assays with TZM-bl cells. The results indicated that the avidity of the Vhh was significantly increased while the neutralization potency was compromised when the Vhh was covalently conjugated to the lipid. The additional ability of the liposome construct to effectively deliver dapivirine, a potent non-nucleoside reverse transcriptase inhibitor presently being developed as a vaginal HIV microbicide, was further investigated.

## Methods

### Materials

Soy phosphatidylcholine (PC), 1,2-dioleoyl-*sn*-glycero-3-[(*N*-(5-amino-1-carboxypentyl)iminodiacetic acid)succinyl] (18:1 DGS-NTA(Ni)) and 1,2-distearoyl-*sn*-glycero-3-phosphoethanolamine-*N*-[maleimide(polyethylene glycol)-2000] (malDSPE-PEG_2000_) were purchased from Avanti Polar Lipids. Methoxypolyethyleneglycol-di-stearoyl-phosphatidylethanolamine (DSPE-PEG2000, with mPEG MW2000Da) was obtained from Genzyme. Cholesterol (Chol), PBS and Sephadex G25 and G50 were purchased from Sigma. Acetone, methanol and dichloromethane were from Fisher Scientific. RPMI1640, l-glutamine, penicillin-streptomycin and foetal bovine serum (FBS) were from Invitrogen Life Technologies. Dulbecco’s minimum essential medium (DMEM) was purchased from Lonza. Vhhs, the variable region fragment of single heavy-chain llama antibodies, including J3 and lab5, were purchased from Quality in Antibodies (Netherlands). Dapivirine, a non-nucleoside reverse transcriptase inhibitor (NNRTI), was kindly provided by the authors Prof. Guido Vanham and Dr. Kevin Arien. CN54 gp140 was kindly provided by Prof. Robin Weiss from University College London.

### Cells, Virus and Reagents

TZM-bl cells (obtained through the NIH AIDS Research and Reference Reagent Program, Germantown, MD) were used for the evaluation of the anti-HIV activity of free and liposome conjugated Vhh with and without dapivirine. TZM-bl cells express high densities of CD4, CCR5 and CXCR4. They contain a luciferase reporter gene under control of HIV LTR, which will be transcribed and translated into luciferase protein, if the cells get infected with HIV and start producing Tat. TZM-bl cells were cultured in DMEM supplemented with 10 % heat-inactivated FBS and 50 μg gentamicin/ml medium, at 37 °C in a humidified 7 % CO_2_ environment. Cells were split twice a week and plated at 10^6^ cells in tissue culture flasks and at 10^4^ cells/well in 96-well plates.

The subtype B CCR-5 using reference HIV-1 strain Ba-L was obtained from the NIH AIDS Research and Reference Reagent Program, Germantown, MD. It was grown and titered in human activated peripheral blood mononuclear cells (PBMC). PBMC were isolated from buffy coats from HIV-seronegative blood donors (provided by the Antwerp Blood Transfusion Centre) using Ficoll density gradient centrifugation. PBMC were stimulated for 48 h with 2 μg/ml of phytohemagglutinin (PHA) (Remel, Kent, UK) in RPMI 1640 medium containing 10 % FBS, 50 μg/ml gentamicin and 2 μg/ml polybrene (Sigma-Aldrich, Bornem, Belgium). Subsequently, PBMC were activated for 24 h with 1 ng/ml of interleukin-2 (IL-2) (Gentaur, Brussels, Belgium) in RPMI 1640 medium supplemented with 10 % FBS, 50 μg/ml gentamicin, 2 μg/ml polybrene and 5 μg/ml hydrocortisone (Calbiochem, Leuven, Belgium). PHA, IL-2 activated PBMC.

### Liposome Preparation

Liposome compositions used in this study with diameters in brackets were as follows: (a) lipo1 (200 nm), PC/Chol/DSPE-PEG_2000_/18:1 DGS-NTA in proportions 65/33/1/1 mol%; (b) lipo2 (100 nm), PC/Chol/DSPE-PEG_2000_/18:1 DGS-NTA in proportions 65/33/1/1 mol% and (c) lipo3 (100 nm), PC/Chol/DSPE-PEG_2000_/malDSPE-PEG_2000_ in proportions 65/33.5/0/1.5 mol%. Briefly, the lipids with or without dapivirine were dissolved in methanol: dichloromethane 1:2 (*v*/*v*) at room temperature. The lipid mixtures were deposited on the side wall of a rotary glass vial by removing the solvent with nitrogen. The resulting dried lipid films were hydrated in 10-mM sodium phosphate buffer pH 7.4. This process led to the spontaneous formation of pegylated liposomes. The liposomes were then extruded through 0.2 or 0.1 μm Anotop 10 filters (Whatman). Excess dapivirine was removed on a Sephadex G25 column [34].

J3 is a neutralizing Vhh, while lab5 is an irrelevant Vhh (irr-Vhh). J3 and lab5 were polyhistidinylated (J3-His and lab5-His) for the non-covalent attachment to liposomes via nitrilotriacetic acid (NTA) linkage. Meanwhile another form of J3 (J3-cys) with extra cysteine in the C-terminus was generated for covalent conjugation to liposomes via interaction with a maleimide group. Vhhs were produced as described before [31]. Non-covalent immunoliposomes were produced by mixing Vhh-His with the liposome suspension in a 1:10 M ratio at room temperature for 1 h on a bench roller. Free thiol groups were generated by incubating J3-cys with 10 mM DTT for 30 min at room temperature. Covalent immunoliposomes were prepared via interaction between J3-cys and maleimide-derivatized lipid inserted in lipo3. The mixture of J3-cys and liposomes with the maleimide group was allowed to conjugate at room temperature overnight. Unreacted maleimide groups were subsequently saturated by adding cysteine. The immunoliposomes were purified by running through a Sephadex G50 column to remove excess cysteine or/and unbound protein.

### Characterization of Liposomes

The phospholipid, mainly PC, in the liposome was quantified by the Stewart assay [34]. The number of liposomes per ml was estimated by assuming there were 80,000 phospholipid molecules per liposome of 100 nm or 320,000 phospholipid molecules per liposome of 200-nm diameter [35]. The mean particle size distribution and polydispersity index of liposome suspensions were determined by dynamic light scattering in a Malvern Zetasizer Nano-2S (Malvern Instruments, Malvern, UK). The buffer for zeta potential is MilliQ water pH 7. The stability of liposomes was evaluated by storing the liposomes at 4 °C for 1 month, and samples were taken for size measurement.

SDS-PAGE was employed to confirm the conjugation of Vhh to the liposomes. Fluorescamine was used to quantify the protein conjugated to liposomes [36]. Fluorescamine is a non-fluorescent compound, which reacts rapidly with primary amines in proteins, such as the terminal amino group of peptides and the ε-amino group of lysine, to form highly fluorescent moieties. Basically, the liposome suspension was diluted 1:20 in PBS pH 7. An aliquot of 25 μl of 2 mg/ml fluorescamine was added to a total assay volume of 100-μl diluted liposome suspension. The fluorescence was measured at ex 390 nm/em 475 nm with a cut-off at 455 nm, and the background was subtracted. The concentration of conjugated protein was calculated by comparison with standard samples of the same protein. The concentration of dapivirine in liposomes was determined by measuring the absorbance at 310 nm after incubating the liposomes in ethanol [37, 38].

### Enzyme-Linked Immunosorbent Assay

An enzyme-linked immunosorbent assay (ELISA) was carried out to determine the interaction between liposome-linked Vhh and gp140 which is a derivative of gp160 that can be cleaved into gp120 and gp41. Briefly, 1 μg/ml CN54 gp140 was coated at 4 °C overnight on 96-well Maxisorp plates (NUNC, Denmark). After blocking with 200 μl/well Microwell Blocking Buffer with Stabilizer (Rockland), serial dilutions of immunoliposomes in PBS pH 7, free Vhh and negative control liposomes coated with irr-Vhh (lab5) were added to the plates for 1 h. After three washes with PBS, anti-myc-horse radish peroxidase (HRP) (Roche), which targets the myc-tag in Vhh, was diluted 1:1000 in HRP Conjugate Stabilizer (Rockland) and added to detect the bound Vhh. 3,3′,5,5′-Tetramethylbenzidine liquid (Sigma) substrate was added and left to develop for 30 min at room temperature; after which, the reaction was stopped with 2 M H_2_SO_4_. Absorbance of the product was measured at 450 nm.

### In Vitro Cytotoxicity

Human cancer cells, Hela cells (Sigma-Aldrich, UK), were cultured in 96-well plates overnight and treated with different concentrations of liposomes for 48 h. Medium was then removed from the plates, and the cells washed twice with PBS. Fifty microlitres of 3-(4,5-dimethylthiazol-2-yl)-2,5-diphenyltetrazolium bromide (MTT) was added per well. Cells were incubated for 30 min at 37 °C with 95 % O_2_ and 5 % CO_2_. MTT was removed carefully before 100-μl propanol was added per well to dissolve crystals and incubated for at least 30 min. The absorbance of this coloured solution was quantified by measuring at a wavelength of 570 nm by FLUOstar Omega (BMG Labtech, Aylesbury, UK). Each treatment was conducted in triplicate.

### Antiviral Assays for Antibody-Mediated Neutralization and Effectiveness of Liposomal Dapivirine Drug Delivery

The antiviral activity of the compounds was determined by pre-incubating 10^4^ TZMbl cells/well in a 96-well plate for 2 h at 37 °C and 7 % CO_2_ to maintain the optimal pH with or without a serial dilution of compounds. Next, 200 TCID_50_ of HIV-1 BaL virus was added to each well, and cultures were incubated for 48 h before luciferase activity was quantified. To this end, 120 μL of supernatant was removed, 75 μl of the luciferase substrate Steadylite (Perkin Elmer, Life Sciences, Zaventem, Belgium) was added to the wells, and the plates were incubated at room temperature on an orbital shaker for 10 min. Next, the luciferase activity was measured using a TriStar LB941 luminometer (Berthold Technologies GmbH & Co. KG., Bad Wildbad, Germany) and expressed in relative light units (RLU).

Each compound was tested in triplicate and each experiment was repeated in three independent runs. Antiviral activity was expressed as the percentage of viral growth compared to the control and plotted against the compound concentration. Next, non-linear regression analysis was used to calculate the EC50_,_ using GraphPad Prism 5.03 using non-linear regression (GraphPad Software, San Diego, CA, USA).

## Results

### Liposome Preparation and Characterization

**Fig. 1:**
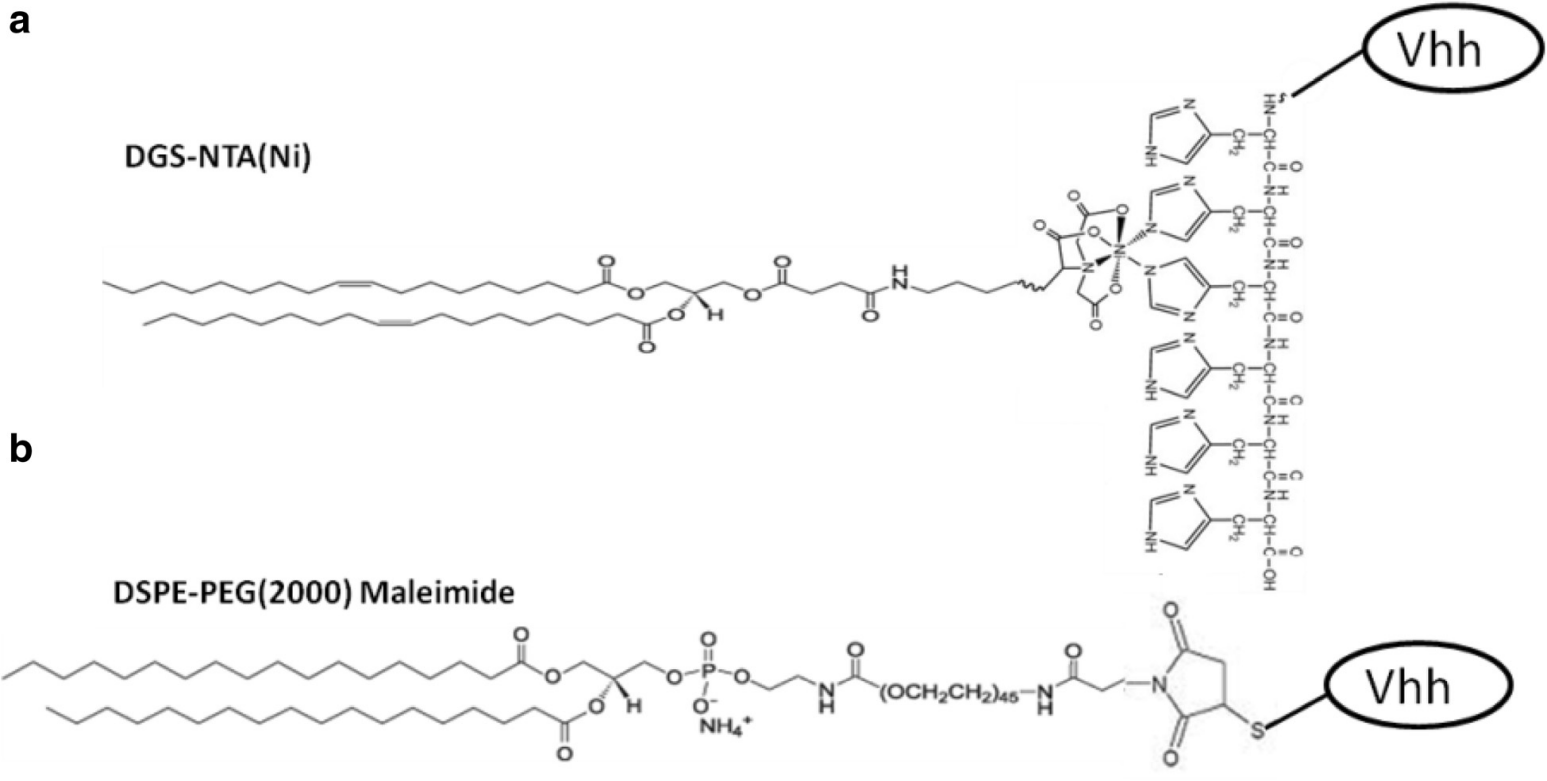
Structural basis of conjugation. **a** Non-covalent ligation between Vhh-His and nickel-chelating lipid 18:1 DGS-NTA-Ni. **b** Covalent ligation between Vhh-cys and malDSPE-PEG_2000_ lipid

**Fig. 2:**
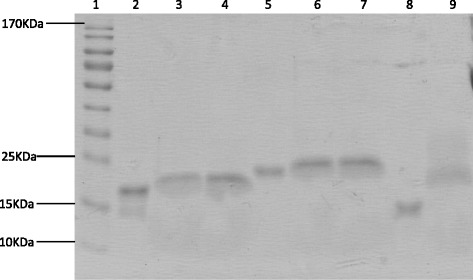
Vhh and conjugated Vhh in liposomes analyzed by SDS-PAGE. Lanes: 1, molecular weight marker; 2, J3-His; 3, lipo1-J3; 4, lipo2-J3; 5, lab5; 6, lipo1-lab5; 7, lipo2-lab5; 8, J3-cys and 9, lipo3-J3

Three groups of liposome suspensions: lipo1, lipo2 and lipo3, were prepared to investigate whether the size or Vhh density of the liposomes will contribute to the neutralization efficiency of the liposomes for HIV virus. Lipo1 was of 200-nm diameter while lipo2 and lipo3 were of 100-nm diameter. His-tagged Vhh (J3-His and lab5-His) were conjugated to lipo1 or lipo2 by interaction with the nickel-chelating lipid 18:1 DGS-NTA-Ni which was incorporated into the liposome bilayer (Fig. 1a). The sulphydryl-reactive lipid (malDSPE-PEG_2000_) in lipo3 allowed covalent attachment of the reduced J3-cys to the liposome (Fig. 1b). Immunoliposomes were then purified by size exclusion chromatography to remove the unbound Vhh and impurities. The conjugation and purification were successful in all cases, and the resulting products were analyzed by SDS-PAGE (Fig. 2). The conjugated Vhhs gave bands of higher molecular weight than the free Vhhs, and this was due to the contribution of the conjugated lipids including 18:1 DGS-NTA-Ni or malDSPE-PEG_2000_. More than 95 % of the Vhh was shown to be conjugated to the liposomes by the fluorescamine method described above [34, 36]. The density of Vhh on liposome surfaces was shown in Table 1.

**Table 1:** Characterisation of the immunoliposomes

Liposome	PC conc. (mg/ml)^a^	Dapivirine (μM)	No. of Vhh molecules per liposome	Vhh (μg/ml)	Diameter (nm)^a^	Zeta potential (mV)^a^
Lipo1-lab5	11.937 ± 0.001	–	160	126.3	228.0 ± 1.0	−45.0 ± 0.9
Lipo1-J3	11.708 ± 0.008	–	160	118.4	223.4 ± 4.5	−37.4 ± 0.5
Lipo2-lab5	5.974 ± 0.005	–	95	150.0	130.9 ± 1.4	−38.7 ± 0.7
Lipo2-J3	6.149 ± 0.003	–	108	159.9	131.2 ± 1.9	−38.5 ± 0.2
Lipo2-dapi	6.478 ± 0.001	6.5	–	–	122.9 ± 1.8	−46.0 ± 0.9
Lipo2-dapi-J3	5.548 ± 0.002	5.5	125	169.6	123.4 ± 1.8	−38.5 ± 0.4
Lipo3 control	7.573 ± 0.003	–	–	–	130.9 ± 1.7	−42.5 ± 0.4
Lipo3-J3	6.456 ± 0.004	–	121	168.2	142.2 ± 3.2	−36.4 ± 0.4
Lipo3-dapi	6.675 ± 0.003	6.7	–	–	128.2 ± 2.6	−41.7 ± 0.5
Lipo3-dapi-J3	6.806 ± 0.001	6.8	108	157.0	139.1 ± 1.8	−37.8 ± 0.6

Dapi: dapivirine

^a^Data are means ± standard deviations of three replicate measurements in one representative experiment of three independent experiments

Immunoliposomes with or without NNRTI dapivirine were produced to investigate whether conjugated Vhh could show higher avidity to antigen and improve the efficacy of Vhh in HIV viral inhibition, as compared to free Vhh. Dapivirine was dissolved in the solvent with lipid mixture when formulating the liposomes. Unincorporated dapivirine was removed by size exclusion chromatography, and the concentration of dapivirine was determined by UV spectrometry.

The final immunoliposomes were characterized, and the data was shown in Table 1. Phospholipid (PC) was detected in the liposomes by Stewart assay. It was estimated to result in approximately 160 Vhh molecules per 200-nm liposome or 95–125 Vhh molecules per 100-nm liposome on the basis that one 100-nm liposome contains 80,000 phospholipid molecules and one 200-nm liposome contains 320,000 phospholipid molecules [29, 35]. This resulted in similar Vhh density on the surface of the liposomes.

**Fig. 3:**
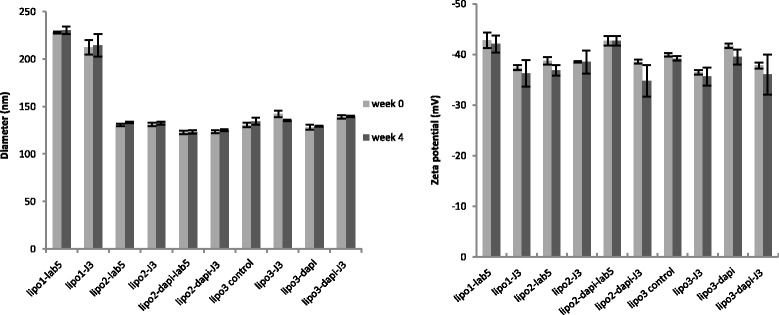
Diameter and zeta potential of the liposome formulations over 1-month period. Data are means ± standard deviations of three replicate measurements in one representative experiment of at least two independent experiments. Using unpaired *t* test (*p* > 0.05), no significant change in diameter or zeta potential of liposomes was observed after 1-month storage at 4 °C. Dapi: dapivirine

PEGylated liposome suspensions were prepared by the lipid film rehydration method [34] and extrusion technique, with a final mean diameter of 200–230 nm or 120–140 nm (polydispersity index of 0.1–0.2). The negative charge of the liposome formulations were attributed to the DSPE-PEG_2000_ or malDSPE-PEG_2000_which accounts for 1–1.5 % mol of the lipid. The liposomes were stable for at least 1 month, with no significant change in size or charge (Fig. 3).

### In Vitro Cytotoxicity of the Liposome

**Fig. 4:**
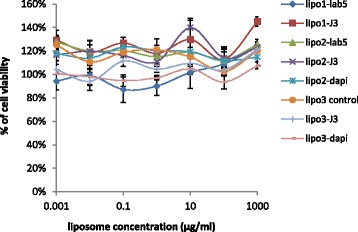
Cell viability of Hela cells after 48 h incubation with control liposomes or immunoliposomes. No significant difference in cell viability was observed against the media only treatment from one-way ANOVA (*p* > 0.05). Data are means ± standard deviations of three replicate measurements in one representative experiment of at least two independent experiments. Dapi: dapivirine

Potential cytotoxicity of the liposomes was estimated from cell viability relative to control Hela cells treated with medium only. MTT assay demonstrated that cell viability was between 80 and 120 % in relation to the control samples (Fig. 4). One-way ANOVA statistical analysis showed there is no evidence of liposome toxicity at concentrations up to 1 mg/ml (*p* > 0.05).

### Analysis of Binding of Liposomal Vhhs to Recombinant HIV gp140 by ELISA

**Fig. 5:**
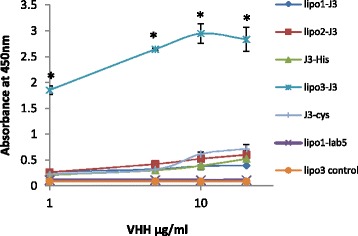
The binding of free J3/J3 linked to liposomes to recombinant gp140 (CN54) was compared in ELISA. Gp140 was immobilized on the plate, and Vhh was detected with anti-myc-HRP. Irrelevant Vhh (lab5) and liposome-irrelevant Vhh were included as negative controls. Values are means ± standard deviations of three replicate measurements in one representative experiment of three independent experiments. *From one-way ANOVA, significant differences were observed (*p* < 0.001) between lipo3-J3 and the other liposome formulations or free Vhhs

As Vhh competed with soluble CD4 (sCD4) for binding to gp120 [32], an ELISA assay was carried out to determine whether the Vhh conjugated to liposomes showed compromised binding affinity or increased avidity to gp140 (CN54). Liposome-linked J3 was compared with the free J3 at the same concentrations, together with the irrelevant Vhh (lab5-His) and liposome-coated the same irrelevant Vhh as negative controls. J3-His and J3-cys revealed similar binding affinities for gp140 as expected (Fig. 5). No improved or compromised affinity was observed for lipo1-J3 and lipo2-J3 compared to free J3-His, implying size differences are not influencing the avidity despite similar Vhh densities on the liposome surfaces. Meanwhile, one-way ANOVA statistical analysis showed that J3 conjugated to lipo3 demonstrated much higher avidity to gp140 than free J3-cys (*p* < 0.001), indicating covalent attachment greatly facilitated J3 interacting with gp140. In contrast, irrelevant negative controls demonstrated no binding to gp140.

### Characterization of Neutralizing Antibody by Antiviral Assay

**Fig. 6:**
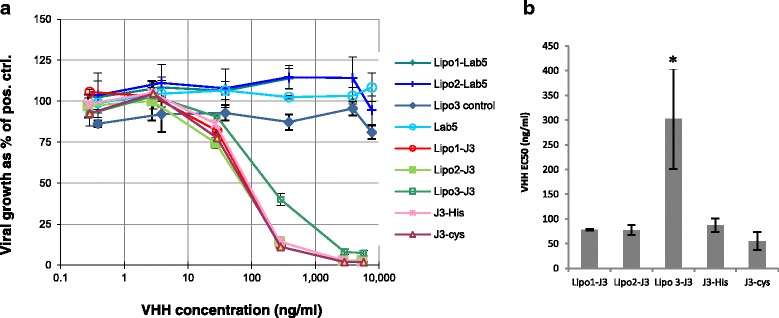
Antiviral effect of different liposomal formulations and free Vhhs. **a** Dose-response curves for the antiviral effect of different liposomal formulations and free Vhhs. **b** Comparison of calculated EC50 of each liposomal formulation and free J3. Liposome preparations were pre-incubated with TZM-bl cells for 2 h before virus was added and incubated for 48 h. Values are means ± standard deviations of three replicate measurements in one representative experiment of three independent experiments. *From one-way ANOVA, significant differences (*p* < 0.001) in EC50 were observed between lipo3-J3 and the other liposome formulations or free Vhhs

The neutralization activity of liposomal Vhhs and free Vhhs for HIV virus was evaluated by subsequent incubation with TZM-bl cells. Lipo1-J3 and lipo2-J3 revealed similar potencies for neutralization compared to free J3. Surprisingly, lipo3-J3 only provided 60 % of viral inhibition at J3 concentration of 286 ng/ml, whereas lipo1-J3 and lipo2-J3 inhibited approximately 90 % of viral growth at the same concentration (Fig. 6a). No viral growth reduction was observed for control liposomes, liposomes coated with irrelevant Vhhs or free Vhhs alone. There was no significant difference in neutralizing activities between lipo1-J3 and lipo2-J3 compared to free J3-His (Fig. 6b), revealing no contribution from the size differences. However, covalently conjugated J3 (lipo3-J3) had a significantly higher EC 50 (Fig. 6b), indicating less potency for neutralization than non-covalently attached J3 or free J3. The result is unexpected because the ELISA data revealed significantly increased binding affinity of lipo3-J3 to gp140 which should contribute to the neutralization activity of conjugated J3.

### Comparison of Antiviral Effects of Liposomal Dapivirine with Free Dapivirine

**Fig. 7:**
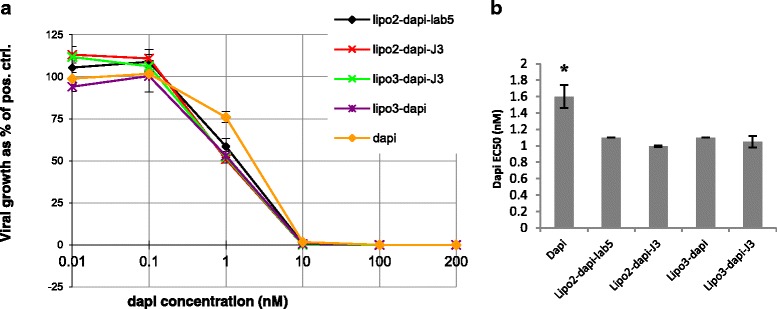
Antiviral effect of different liposomal formulations and free dapivirine. **a** Dose-response curves for the antiviral effect of different liposomal formulations and free dapivirine. **b** Comparison of calculated EC50 of each liposomal formulation and free dapivirine. Liposome preparations were pre-incubated with TZM-bl cells for 2 h before virus was added and incubated for 48 h. Values are means ± standard deviations of three replicate measurements in one representative experiment of three independent experiments. *From one-way ANOVA, significant differences (*p* < 0.001) in EC50 were observed between free dapivirine and the liposomal dapivirine. Dapi: dapivirine

We compared the antiviral effects of different formulations of liposomal dapivirine with free dapivirine. The dose-response curves indicated increased antiviral effect of liposomal dapivirine compared with free dapivirine, although no significant difference was observed between lipo2 and lipo3 with or without neutralizing Vhhs (Fig. 7a). Furthermore, there was approximately 76 % of viral growth at a concentration of 1-nM free dapivirine, whereas only 50–56 % of viral growth was detected with liposomal dapivirine at this concentration. Calculated EC50 (Fig. 7b) confirmed that liposomal dapivirine is more effective than free dapivirine in inhibiting HIV virus. However, the antiviral effects were dominated by dapivirine since no difference was observed between the liposome preparations with or without neutralizing J3.

## Discussion

The treatment of HIV remains a serious challenge due to the high genetic variation of the virus and associated toxic side effects of the antiviral drugs. HIV-1 neutralizing antibodies have been considered to be critical for vaccine development and prevention of HIV infection. Liposomes provided another approach shown to have potential for HIV prophylaxis [39]. In the present study, a combined platform with both liposome and antibody fragments was developed to investigate the antiviral efficiency of the multivalent liposomal formulation.

Previous studies have demonstrated that Vhhs produced by llamas (*lama glama*) exhibited comparable affinity and specificity for gp120 to conventional immunoglobulins despite their much smaller size. This smaller size of Vhhs is an advantage over conventional immunoglobulins as it has been shown to reduce their immunogenicity [33]. In particular, J3 Vhh, which achieved broad and potent neutralization of HIV-1 via interaction with the CD4-binding site, neutralized 96 of 100 tested HIV-1 strains and chimeric simian-HIV strains with HIV subtypes B and C env [33]. Unlike most of the enveloped viruses which express large numbers of closely spaced spikes on the surface, HIV virus has a low spike surface density (4 to 35 spikes) and the arrangement of these spikes is likely to be static over the time periods relevant to neutralization [14]. Therefore, the location and arrangement of the neutralizing Vhhs on the liposome surfaces is critical for their interaction with the virus particles.

To optimize the size of the liposomal platform, J3 was non-covalently conjugated to the surface of two liposome formulations with diameters of 100 or 200 nm, each with approximately the same Vhh densities. Metal chelation via NTA has been employed to attach peptide and protein antigens to liposomes to compare with covalent linkage for their ability to elicit antibody responses [40]. Pegylated liposomes were employed to ensure the stability and minimal immunogenicity of the platform. To check that J3 was available and not masked by the PEG coating on the liposome surface, appropriate access of J3 to gp140 was verified by ELISA (Fig. 5). The identical binding curves demonstrated that lipo1-J3 and lipo2-J3 bound to gp140 with the same avidity as free J3, showing that the different geometrical location of J3 on the surface of spherical liposomes did not compromise its interaction with gp140. Surprisingly, no enhanced avidity was observed for these liposomal J3 constructs compared to free J3. This may be due to the charge on the nickel-histidine complex which would inhibit close association of the J3 antibody fragments to generate multivalent attachment to the gp140 on ELISA surface. This presumption is confirmed by the neutralization results indicating lipo1-J3 and lipo2-J3 inhibited HIV infection in a dose-response manner similar to free J3 in antiviral assay (Fig. 6). There was no significant difference in the EC50 between lipo1-J3, lipo2-J3 and free J3, suggesting no improved antiviral potency for the non-covalently anchored Vhh independent of liposome sizes.

J3-cys was then covalently attached to liposomes to produce lipo3-J3 (Fig. 1). One hundred-nanometre diameter liposomes were used for economic reasons and since liposomes of this size are broadly applied as drug delivery system [41, 42]. The binding curves for lipo2-J3 and lipo3-J3 were distinct from each other, indicating that their interactions with gp140 were qualitatively different with superior avidity for lipo3-J3. This implied that a multivalent platform had been formed with cross-linking or multimerization of Vhhs. This is not surprising since the absence of the nickel complex allows the free movement and geometrical arrangement of J3-lipid complex within the lipid liposome membrane so permitting the formation of multivalent liposomal Vhh constructs through cross-linking the envelope spikes which are sparsely distributed on the virion surface and remained static over the time periods. Unexpectedly, the antiviral potency of lipo3-J3 was remarkably reduced as evaluated in the antiviral assay compared to lipo1-J3, lipo2-J3 or free J3, the EC50 for lipo3-J3 being approximately four times higher than that for lipo2-J3 or free J3. We hypothesize that in the case of non-covalent linkage, J3 was dissociated from liposome after it bound to gp140 on the virion surface because the interaction between J3 and gp140 was so strong (*K* ≅ 10^−9^ M ~ 10^−12^ M compared to *K* ≅ 10^−5^ M ~ 10^−6^ M for attachment to the nickel-chelating lipid) [32, 40, 43], that J3 was pulled apart from the nickel complex inserted in the liposome membrane. The remaining surface antibody molecules were now free to react with further virus, the net effect being that lipo2-J3 neutralized the virus as efficiently as free J3. Although the covalent attachment of the J3 in lipo3 enhanced lipo3-J3-gp140 interaction, this fixed the viruses to the surface of the liposome and due to the comparability in size of the interacting particles, the number bound to the liposome surface will be restricted and the small number of viral spikes involved will leave a large fraction of the antibodies remaining uncombined which were otherwise available in the non-covalently linked constructs.

In addition to the neutralization capacity of antibody-coated liposomes, we investigated whether the liposomes could have the extra-function of delivering antiviral drugs intracellularly, in particular using high doses of otherwise poorly soluble hydrophobic drugs whose encapsulation in the liposome would limit its general toxicity. All of these dapivirine containing liposomes revealed higher efficacy in reducing viral replication than free dapivirine, although no significant differences were observed between them i.e. irrespective of the presence of irrelevant or J3 Vhh on the surface. This suggested that the antiviral activities of J3 were masked by the high dose of dapivirine and that the liposome platform per se is an efficient delivery system for dapivirine.

## Conclusions

This study demonstrated that antibody-conjugated liposomes can provide anti-HIV viral defence in two ways. First, non-covalently conjugated liposomal J3 neutralized HIV virus as efficiently as free J3. It was also shown that incorporation of the lipophilic drug dapivirine in liposomes resulted in a higher level of virus inactivation in vitro than observed after exposure of cells to free drug dissolved in DMSO—an effective method of solubilizing the drug, but not one which can be used in vivo. Since we have demonstrated that immunoliposomes with covalently grafted anti-CD4 Vhhs facilitated cellular uptake of liposomes by lymphoma cells overexpressing CD4 on the surface (data not shown), it follows that a combined liposome platform encapsulating dapivirine with non-covalently attached J3 and covalently linked anti-CD4 Vhh could be a powerful potential candidate for HIV prevention where J3 neutralizes free virus and dissociates from liposome while the anti-CD4 Vhh coated liposome efficiently delivers the antiviral drug to HIV target cells overexpressing CD4.
